# Furfuralcohol Co-Polymerized Urea Formaldehyde Resin-derived N-Doped Microporous Carbon for CO_2_ Capture

**DOI:** 10.1186/s11671-015-1041-x

**Published:** 2015-08-21

**Authors:** Zhen Liu, Yi Yang, Zhenyu Du, Wei Xing, Sridhar Komarneni, Zhongdong Zhang, Xionghou Gao, Zifeng Yan

**Affiliations:** State Key Laboratory of Heavy Oil Processing; Key Laboratory of Catalysis, CNPC, China University of Petroleum, Qingdao, 266580 PR China; Materials Research Institute, Pennsylvania State University, University Park, PA 16802 USA; Lanzhou Petrochemical Research Center, Petrochemical Research Institute, PetroChina Company Limited, Lanzhou, 730060 PR China

**Keywords:** N-doped, Microporous carbon, High N-containing, Fine micropores, CO_2_ capture

## Abstract

Carbon-based adsorbent is considered to be one of the most promising adsorbents for CO_2_ capture form flue gases. In this study, a series of N-doped microporous carbon materials were synthesized from low cost and widely available urea formaldehyde resin co-polymerized with furfuralcohol. These N-doped microporous carbons showed tunable surface area in the range of 416–2273 m^2^ g^−1^ with narrow pore size distribution within less than 1 nm and a high density of the basic N functional groups (2.93–13.92 %). Compared with the carbon obtained from urea resin, the addition of furfuralcohol apparently changed the surface chemical composition and pore size distribution, especially ultramicropores as can be deduced from the X-ray photoelectron spectroscopy (XPS), Fourier transform infrared (FT-IR), and pore size distribution measurements and led to remarkable improvement on CO_2_ adsorption capacity. At 1 atm, N-doped carbons activated at 600 °C with KOH/UFFC weight ratio of 2 (UFFA-2-600) showed the highest CO_2_ uptake of 3.76 and 1.57 mmol g^−1^ at 25 and 75 °C, respectively.

## Background

Carbon dioxide as the main contributor to global warming has attracted extensive attention worldwide. The reduction or elimination of CO_2_ emission into the atmosphere is extremely urgent. Therefore, carbon capture and storage (CCS) has been a research hotspot in recent years. Among various technologies for CCS, adsorption is considered to be one of the most promising techniques in practical application due to low energy consumption and mild operating conditions compared to solvent absorption, membrane systems, and cryogenic fractionation [1]. It is possible to reach the goal of the US Department of Energy (DOE) to develop a fossil fuel conversion system capable of capturing 90 % of the produced CO_2_ that increases the total costs by less than 10 % [2]. However, high adsorption capacity, high CO_2_/N_2_ selectivity, and outstanding cycling performance are the key essentials for pressure-swing adsorption or temperature-swing adsorption technology [3]. In this context, zeolites [4], porous carbons [5], porous silicas [6], BCN graphene analogues [7], porous organic polymers [8], MOFs [9, 10], and COFs [11, 12] have been studied for CO_2_ adsorption in recent years. Compared with other materials, carbonaceous adsorbent possesses unique superiority in terms of capture capacity and selectivity due to its hydrophobic property, high thermal and chemical stability, tunable pore structure, low cost, and physical adsorption mechanism [13].

Recently, researchers [14–16] have shown that the fine micropores and heteroatom incorporation are the decisive factors, which affect adsorption properties. N-doped carbon material with proper pore structure, owing to the promotion of the interaction between CO_2_ molecules and carbon surface, is considered to be more efficient for CO_2_ adsorption than traditional carbon such as commercial activated carbon with low capacity of 0.89 mmol g^−1^ and low adsorptive selectivity [17]. The CO_2_ uptake of N-doped carbon material can be enhanced effectively so that it can be close to the minimum working capacity required to match the efficiency of a conventional liquid-phase amine-based system [18]. For example, Lu et al. [16] synthesized N-doped porous carbon monolith with the copolymer of resorcinol, formaldehyde, and lysine as precursor which has shown high CO_2_ capacity of 3.13 mmol g^−1^ at 25 °C, 1 atm.

Besides the effect of nitrogen incorporation, the presence of ultramicropores is considered to be another decisive factor. Recently, Presser et al. [19] studied the CO_2_ uptake on microporous carbon in relation to the pore size and found that the micropores smaller than 1 nm are responsible for high CO_2_ adsorption at 1 bar. Jaroniec and Wickramaratne [20, 21] obtained phenolic resin-based carbon with unprecedented amount of CO_2_ (4.6 mmol g^−1^ at 23 °C), and their study also showed that a higher capacity resulted from a higher pore volume of fine micropores (<0.8 nm). Zhao et al. [22] prepared N-doped carbon material using *p*-diaminobenzene as precursor and found that furfuralcohol can improve the adsorption effectively, but the mechanism is not elaborated clearly. From our point of view, we are trying to find a method for the fabrication of N-doped carbon material with high CO_2_ capture capacity from widely provided low-cost raw chemicals. In our previous work, we found that urea formaldehyde resin had good ability to produce carbon material with high CO_2_ capture capacity [23]. Then, we expanded our strategy to urea furfural resin polymerized from urea and furfural, which also showed high CO_2_ adsorption capacity [24]. However, the carbon source of furfural is not very widely provided as formaldehyde. Here, in this study, for the purpose of further improving the CO_2_ adsorption capacity, furfuralcohol was chosen to form a copolymer with urea and formaldehyde and treated by the same carbonization-activation process to make porous carbon. The samples co-polymerized with furfuralcohol showed remarkable improvement of CO_2_ adsorption capacity mainly due to the development of fine micropores, even though they possess comparable specific surface area (about 1000 m^2^/g) and lower nitrogen content, suggesting that the fine micropores, especially ultramicropores, play a more important role in enhancing the CO_2_ adsorption capacity of adsorbents.

## Method

### Materials Preparation

The samples were prepared by a two-step method. For a typical preparation, 3.0-g urea was added to 4.1 g formaldehyde solution and stirred for 0.5 h, then a certain amount of furfuralcohol (furfuralcohol/urea at a weight of 0, 1, 1.5, and 2) was added to the solution and stirred for 1 h, and then polymerized at 373 K for 24 h. The obtained furfural resin (UFF) was carbonized at 873 K for 4 h under a N_2_ flow of 50 ml min^−1^. The carbonized sample (denoted as UFFC) was chemically activated with KOH (KOH/UFFC at a weight ratio of 1 to 4) at several temperatures in the range of 500–800 **°**C with a ramping rate of 5 **°**C min^−1^ under N_2_ flow of 50 ml min^−1^ and maintained for 1 h. The obtained material was washed with excess amounts of 1 M HCl aqueous solution followed by deionized water till a neutral pH. Finally, the samples were dried in air at 100 **°**C for 12 h. The activated carbon was denoted as UFFA*a*-*x*-*y*, where *a* represents the furfuralcohol/urea weight ratio and be omitted when furfuralcohol/urea weight ratio is 1.5, and *x* and *y* represent the KOH/UFFC weight ratio and activation temperature, respectively.

### Materials Characterization

The morphologies of the materials were examined by scanning electron microcopy (SEM) using a HITACHI S-4800 and transmission electron microscopy (TEM) using a JEOL JEM-2100UHR transmission electron microscope. X-ray diffraction patterns were recorded in the range of 2*θ* = 10°–80° on a PANalytical X’Pert PRO MPD, using a Cu-Kα monochromated radiation source and a Ni filter. The nitrogen adsorption-desorption isotherms were measured at −196 °C using a Micromeritics Tristar 3000 apparatus. Prior to measurement, the samples were degassed in vacuum at 300 **°**C for at least 3 h. The total specific surface area was calculated using the Brunauer-Emmett-Teller method (*p*/*p*_o_ = 0.05–0.25), and the microporous surface area was calculated by the *t*-Plot method. Narrow micropore distribution was analyzed on Micromeritics ASAP 2020. The micropore distributions were calculated via the Horvath-Kawazoe (HK) method. Fourier transform infrared (FT-IR) spectra were obtained on a Nicolet Fourier spectrophotometer, using the KBr pellet technique. The contents of C, H, and N were determined by *using* a Vario EL III CHNS/O elemental analyzer. X-ray photoelectron spectroscopy (XPS) measurements were carried out in a UHV system using a monochromated Al K*α* radiation (h*ν* = 1486.6 eV) and an Omicron Sphera II hemispherical electron energy analyzer.

### CO_2_ Capture Measurements

CO_2_ adsorption was performed on Micromeritics Tristar 3000 apparatus at different temperatures (25 and 75 °C). Prior to the analysis, the samples were degassed at 300 °C in vacuum for 4 h. The adsorption capacity was investigated by exposing quantitative amount of CO_2_ with a concentration of 99.99 % and calculated in terms of adsorbed amount per gram of sample in the pressure range between 0.001 and 1 atm.

## Results and Discussion

### Structural Characterization

**Fig. 1:**
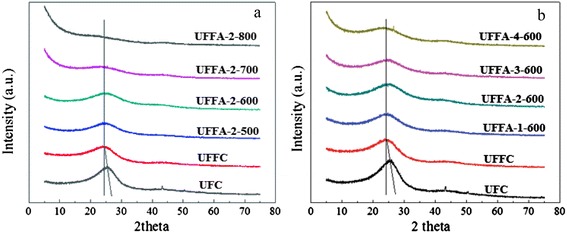
XRD analysis of different samples. **a** Samples activated at different temperatures. **b** Samples activated with different amounts of KOH

In our previous work [23, 24], as well as the previously reported literature [25], it has been demonstrated that the carbonization-activation process played an important role in controlling the nitrogen content and pore structure of the material. Here, two batches of porous carbon materials with different activation temperatures and amounts of KOH addition were synthesized, and their wide angle XRD patterns are shown in Fig. 1. All samples exhibit a broad diffraction peak at about 25.8°, which can be assigned to diffraction from (002) corresponding to *d* spacing of 3.42 Å. This indicates turbostratic ordering of the atoms in the graphene layers of the samples [26]. With the increase of the activation temperature or the amount of KOH, the intensity of (002) reflection decreases noticeably, indicating that a higher activation temperature or excess KOH could destroy the graphite-like structure. The structure of the sample appears to be more sensitive to the activation temperature. No obvious diffraction peak can be detected when the sample was activated at 800 °C (UFFA-2-800 in Fig. 1a), which demonstrates that amorphous carbons were obtained. Additionally, compared to the sample without furfuralcohol addition (UFC), the diffraction peaks of UFFC samples shift slightly to low-angle region, which demonstrates that the interlayer d spacing gets larger. This phenomenon may have resulted from the disordering induced by the addition of furfuralcohol, which may benefit the formation of micropores during the activation process.

**Fig. 2:**
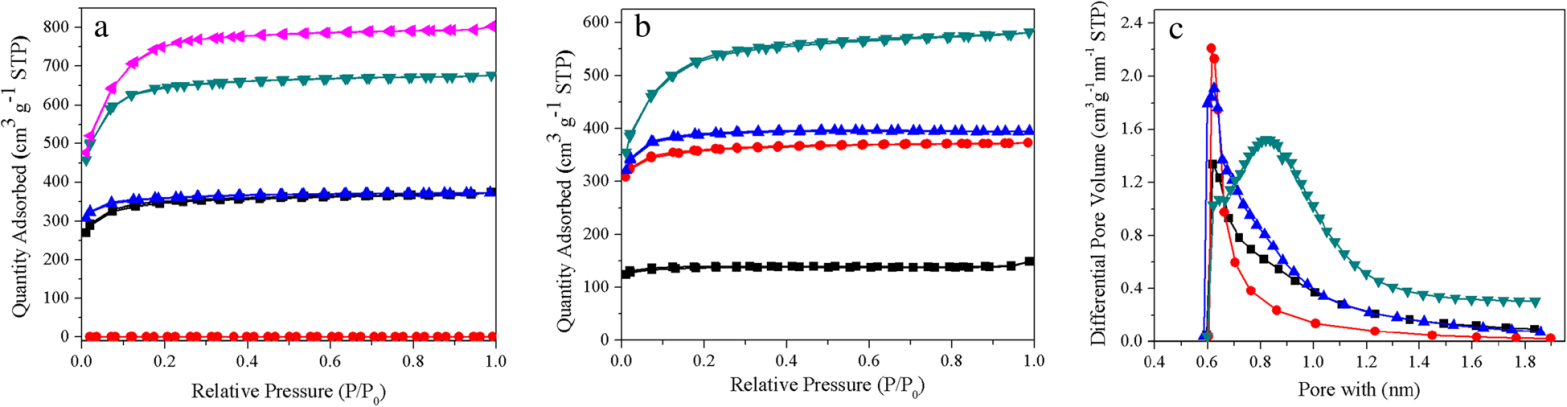
N_2_ isotherms and micropore distributions of typical samples. **a** Samples activated at different temperatures (*black square* UFA-2-600, *red circle* UFFA-2-500, *blue up-triangle* UFFA-2-600, *dark cyan down-triangle* UFFA-2-700, *magenta left-triangle* UFFA-2-800). **b** Samples activated with different amounts of KOH (*black square* UFFA-1-600, *red circle* UFFA-2-600, *blue up-triangle* UFFA-3-600, *dark cyan down-triangle* UFFA-4-600). **c** Micropore distribution of typical samples investigated by N_2_ adsorption at low relative pressure of below 0.03 (*black square* UFA-2-600, *red circle* UFFA-1-600, *blue up-triangle* UFFA-2-600, *dark cyan down-triangle* UFFA-2-800)

In order to investigate the textural properties, the samples were characterized by the nitrogen adsorption-desorption analysis. The isotherms of the obtained materials at different activation temperatures and different amounts of KOH are displayed in Fig. 2. All isotherms are typical type I isotherms with significant N_2_ uptake at low relative pressure (*P*/*P*_O_ < 0.1) and an adsorption plateau at higher relative pressure. All these features confirm that the obtained materials are highly microporous except for UFFA-2-500. The sample activated at 500 °C showed an extremely low specific surface area as 28 m^2^ g^−1^, which indicates that the sample was lack of activation at such low temperature. Actually, the sample before activation (UFFC) showed even much lower specific surface area (0.2 m^2^ g^−1^) than UFFA-2-500. After the activation temperature increased up to 600 °C, the specific surface area increased remarkably, which means that chemical reaction of KOH with raw carbon material was accelerated. Furthermore, no hysteresis loops are present, which indicates that no mesopores are present in the materials. Compared with UFA-2-600, the sample UFFA-2-600 obtained from the same carbonization and activation treatment showed almost the same N_2_ adsorption-desorption isotherms indicating a comparable Brunauer-Emmett-Teller (BET) surface area and pore volume (1055 m^2^ g^−1^ and 1093 m^2^ g^−1^ with the same pore volume of 0.58 cm^3^ g^−1^, Table 1), and the samples carbonized with different furfuralcohol dosages also showed comparable BET surface area and pore volume indicating that the impact of furfuralcohol on the total surface area and pore volume is not significant. However, the microporous specific surface area and microporous pore volume of UFFA-2-600 is much higher than that of the sample without furfuralcohol addition (955 m^2^ g^−1^ and 0.49 cm^3^ g^−1^ versus 861 m^2^ g^−1^ and 0.45 cm^3^ g^−1^, Table 1). Moreover, the features of isotherms are changed with different activation temperatures and amounts of KOH indicating changes of the surface area and pore structures. With the increase of the activation temperature and KOH dosage, the surface area and pore volume increased significantly from 416 to 2273 m^2^ g^−1^ and 0.23 to 1.24 cm^3^ g^−1^, respectively (Table 1). It is worth noting that most of the pore volume can be ascribed to micropore volume, which confirms that the micropores are dominant. The sample prepared under mild activating condition (UFFA-2-600) possesses the highest proportion of micropore volume (0.49 cm^3^ g^−1^/0.58 cm^3^ g^−1^). Further increase of the activation temperature (UFFA-2-800) or KOH dosage (UFFA-4-600) led to a decrease of the proportion of micropore volume. This result indicates that the activation process can not only lead to high porosity but also affects the pore structure.

**Table 1:** Textual properties and chemical composition of various samples

Sample	Textual properties						Chemical composition by CHN			
	*S*_Total_	*S*_Micro_	*S*_Meso_	*V*_Total_^a^	*V*_Micro_	*V*_Meso_^b^	H	C	N	O^c^
	m^2^ g^−1^	m^2^ g^−1^	m^2^ g^−1^	cm^3^ g^−1^	cm^3^ g^−1^	cm^3^ g^−1^	wt %	wt %	wt %	wt %
UFC	5.13	–	5.13	0.0022	–	0.0022	1.85	66.15	23.73	8.27
UFFC	0.2	–	–	–	–	–	2.31	74.84	13.92	8.93
UFA-2-600	1055	861	194	0.58	0.45	0.13	1.77	65.02	13.87	19.34
UFFA1-2-600	1054	931	123	0.55	0.48	0.06	2.95	67.18	7.29	22.58
UFFA-2-600	1093	955	138	0.58	0.49	0.09	2.83	66.96	8.03	22.18
UFFA2-2-600	1000	871	129	0.53	0.45	0.07	2.86	66.01	8.46	22.67
UFFA-1-600	416	384	32	0.23	0.2	0.03	3.23	68.32	10.00	18.45
UFFA-3-600	1175	1043	132	0.61	0.54	0.05	3.30	67.32	7.76	24.62
UFFA-4-600	1639	880	759	0.90	0.48	0.42	2.45	78.3	5.18	14.07
UFFA-2-500	28	19	9	0.03	0.01	0.02	3.32	63.27	10.33	23.08
UFFA-2-700	1925	1456	469	1.04	0.79	0.25	2.03	79.82	6.25	11.9
UFFA-2-800	2273	1248	1025	1.24	0.70	0.54	0.72	89.08	2.93	7.27

*S*_Total_, *S*_Micro_, and *S*_Meso_ are calculated by BET analysis, *t*-Plot analysis, and *S*_BET_-*S*_Micro_, respectively

^a^Total pore volume of pores at *P*/*P*o = 0.99

^b^ *V*_Meso_ is calculated by subtracting *V*_Total_ to *V*_Micro_

^c^Calculated by subtracting CHN content

The narrow pore size distribution of selected samples was further investigated by N_2_ adsorption at low relative pressure of below 0.03, which is shown in Fig. 2c. It should be noted that the samples carbonized with furfuralcohol and activated in mild conditions generated much more micropores centered at around 0.6 nm than UFA-600. Additionally, the pore size is highly dependent on activated conditions with an obvious enlargement of the pore size. The sample activated at 800 °C possessed enlarged micropores of about 0.9 nm and a broadening of the pore size distribution. This pore enlargement is probably due to the over-activation at higher temperature and is consistent with aforementioned results [23], as well as the literature reported [27].

**Fig. 3:**
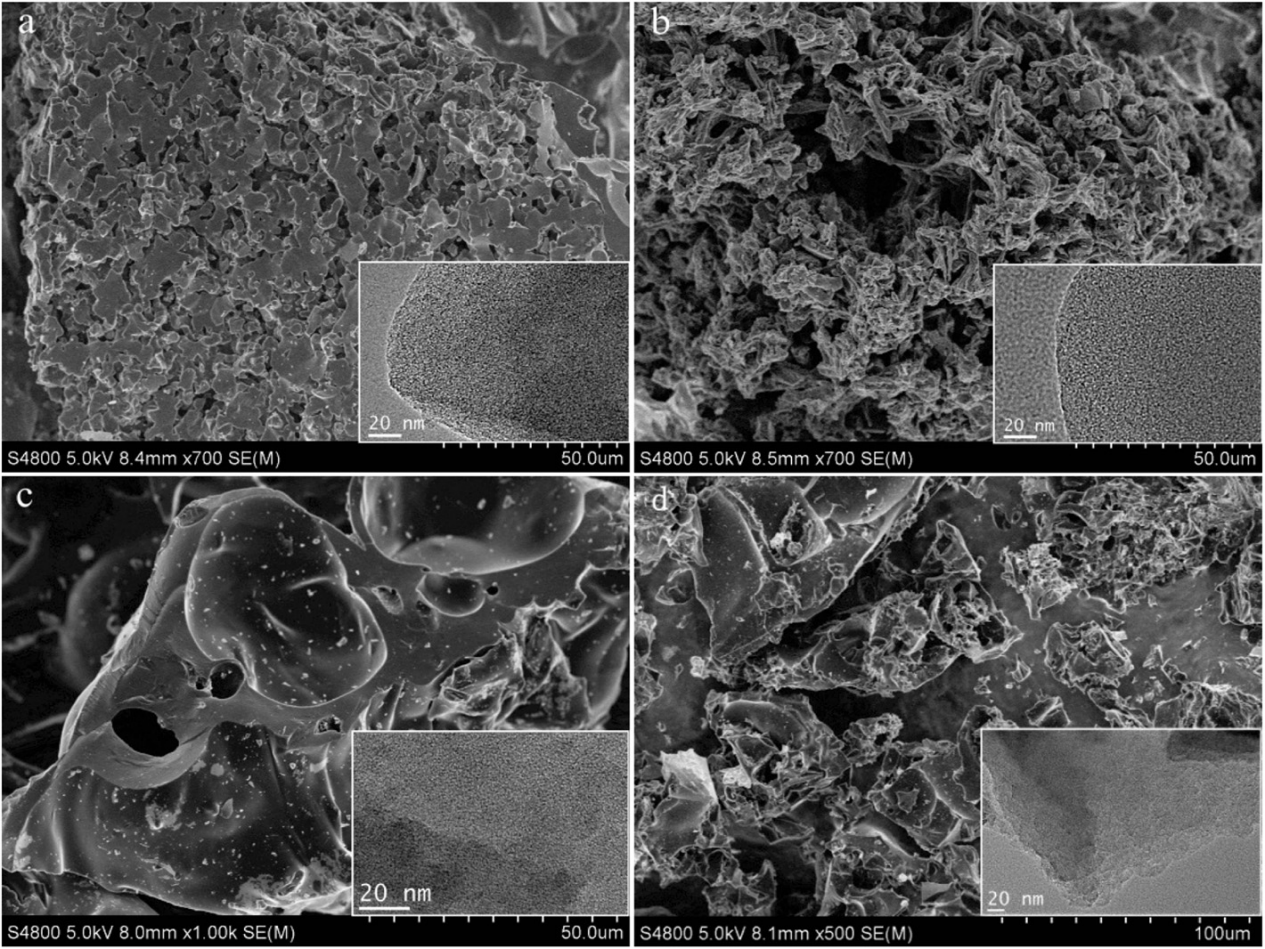
SEM images of carbon materials prepared with or without furfuralcohol addition. **a** UFFC; **b** UFFA-2-600; **c** UFC, and **d** UFA-2-600; the *insets* are the corresponding TEM images

In order to reveal the morphology of the samples, scanning electron microcopy (SEM) and transmission electron microscopy (TEM) were used and the morphology of the obtained materials is shown in Fig. 3. UFFC before activation possessed 3-D net shape of macroporous structure as shown in Fig. 3a, which may have been inherited from the morphology of the furfural resin, and is benefited by mixing with KOH during activation. In contrast, the sample prepared without furfuralcohol (UFC) displays bulk morphology with no obvious mesopores or macropores (Fig. 3c). Both of the TEM images demonstrate that some worm-like micropores were formed by stacking of curved graphene in the carbonized samples (UFC and UFFC). These considerably fine micropores cannot be characterized by N_2_ adsorption-desorption isotherms due to the ultrafine nature. After activation, it can be observed from the SEM image that the 3-D net shape of macroporous structure was preserved (Fig. 3b), but the bulk morphology gets spongier. Many more micropores were also formed during the activation process. It is postulated that the micro-macro hierarchical pore structure should benefit CO_2_ adsorption and desorption due to reduced mass transfer resistance.

### Chemical Properties

The chemical compositions of the obtained samples are listed in Table 1, and it can be seen that higher nitrogen content was still retained even after activating under severe conditions. In contrast to UFA, the nitrogen content of the UFFA samples with furfuralcohol addition was decreased due to higher proportion of carbon source of the precursor. Interestingly, an increase in the nitrogen content is observed with the increase of the dosage of furfuralcohol, from 7.29 to 8.46 %. One possibility is that KOH prefers etching the fragments carbonized from furfuralcohol to that from urea resin, and the nitrogen atoms are protected during the activation process resulting from different surface charge densities [22]. What is noteworthy is that the insertion of O atoms into the framework occurs during the activation process resulting in the increase of the oxygen content. It is well known that the increase of the activation temperature or the dosage of KOH leads to the release of nitrogen in the form of molecular N_2_, HCN, or other gases [28], which is responsible for the decreased of nitrogen content. However, the release of nitrogen reduces the affinity of the samples to CO_2_ molecule and this can be confirmed by the CO_2_ adsorption capacity and the isosteric heat of adsorption, which will be elaborated latter.

**Fig. 4:**
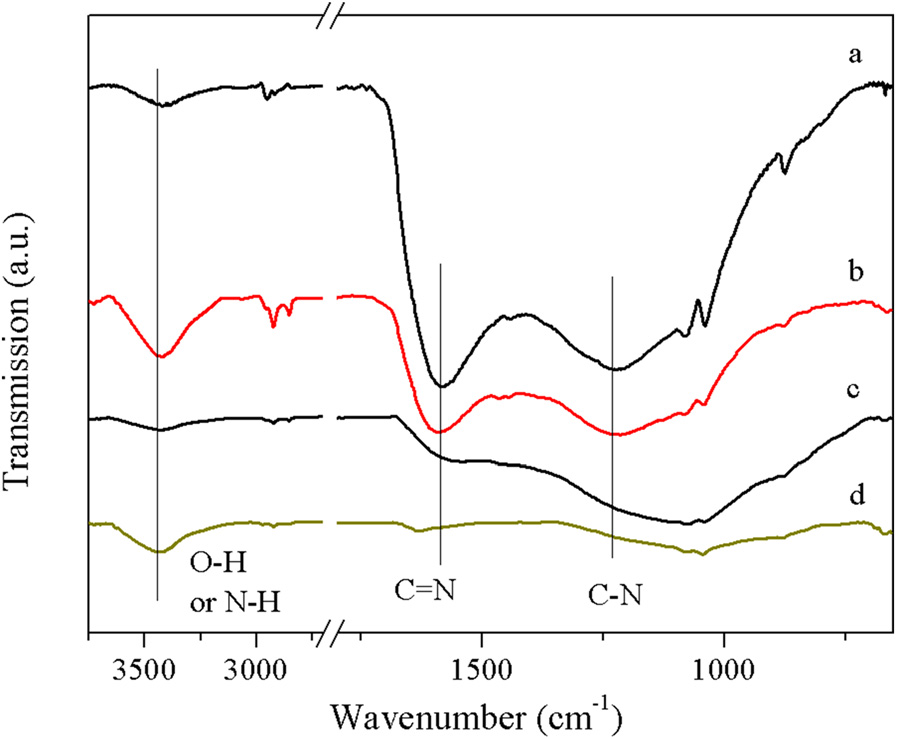
FT-IR spectra of typical activated samples. UFA-2-600 (*a*), UFFA-2-600 (*b*), UFFA-4-600 (*c*), and UFFA-2-800 (*d*)

The previous researches [13, 29, 30] demonstrated that the N-containing functional groups are closely related to the CO_2_ adsorption capacity of the adsorbent. Accordingly, the presence of nitrogen functionalities in these as-synthesized carbon materials was investigated by means of FT-IR. Figure 4 shows the FT-IR spectra of the samples with different activation temperatures and KOH dosages. The bands of the activated samples get broader and overlap due to the strong adsorption of carbons. Notably, the sample UFA-2-600 possesses similar bands with only slight intensity differences to the samples prepared with furfuralcohol addition, demonstrating that the addition of furfuralcohol did not change the surface chemical property significantly. Generally, all the activated samples exhibit two medium bands at around 1590 and 1250 cm^−1^, which can be assigned to C═C stretching vibrations in aromatic rings and C–N stretching vibrations, respectively. Two bands at ca. 2940 and 2828 cm^−1^ are attributed to the stretching vibration of methylene and the peak at 3410 cm^−1^ corresponds to the N–H or O–H symmetric stretching vibration [16]. Three sharp bands are also detected at 1080, 1039, and 875 cm^−1^, respectively, which might be assigned to asymmetric stretching vibration of the ether, C–O stretch of C–OH, and cyclic ether linkages, respectively. All of the FT-IR analysis results confirm the existence of C–N and C–O species in the obtained materials. In addition, the intensity of the band at 1250 cm^−1^ decreased with the increase of the activation. The band of UFFA-2-800 at 1250 cm^−1^ almost disappears when activated at 800 °C, demonstrating that the nitrogen atoms were gradually removed. This result is also confirmed by the elemental analysis.

**Fig. 5:**
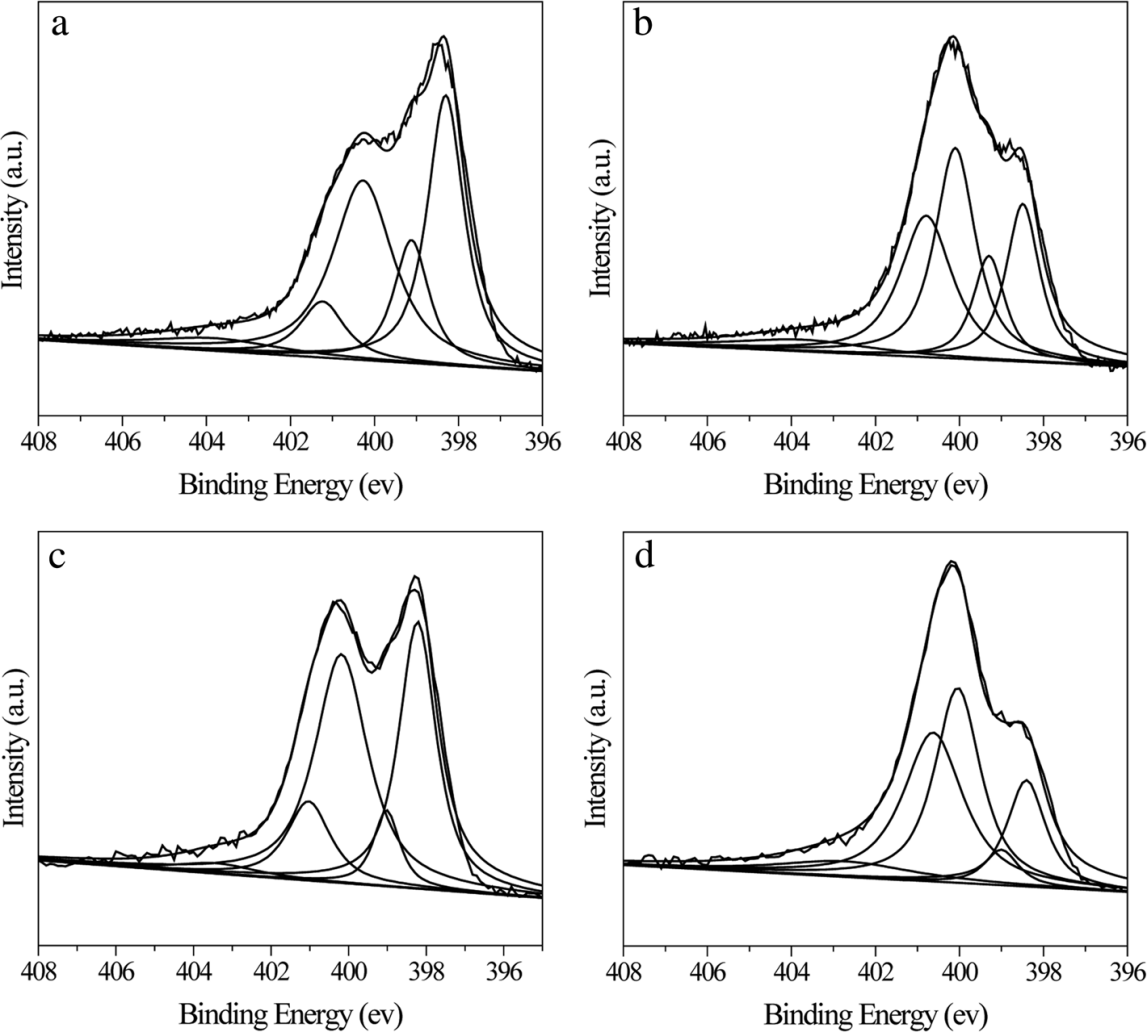
Peak-fitting of XPS N1s signals of selected samples. Carbonized UFC (**a**) and UFFC (**c**) and the activated UFA-2-600 (**b**) and UFFA-2-600 (**d**)

The nature of nitrogen species was further investigated by X-ray photoelectron spectroscopy (XPS). In Fig. 5, the fitting of N1s peak gives the binding energies of 398, 399, 400, 401, and 403 eV, which can be attributed to pyridinic-N, -C═NH or PhNH_2_, pyrrolic-N, quaternary-N, and oxidized-N [31], respectively. The peak assignment reveals that the nitrogen species evolution occurred before and after activation as well as by the addition of furfuralcohol. As shown in Table 2, much more nitrogen species in UFFC are in the form of nitrogen-containing heterocyclic compounds (pyridinic/pyrrolic-N and quaternary-N) than those in UFC (90 to 79.8 %), which is obviously induced by the addition of furfuralcohol. This result confirms our inference that nitrogen species are in more stable heterocyclic form when carbonized with furfuralcohol. After the carbon being activated, a significant increase of quaternary-N was observed, which was mainly transformed from pyridinic-N, -C═NH or PhNH_2_, and pyrrolic-N. The total N content of the surface also presents obvious decrease due to activation process, which is also in accordance with the results of FT-IR and elemental analysis.

**Table 2:** N-containing groups on the surface of the prepared carbons

Sample	Pyridinic-N (%)	-C═NH PhNH_2_ (%)	Pyrrolic-N (%)	Quaternary-N (%)	Oxidized-N (%)
UFC	34.1	14.7	37.2	8.5	5.5
UFA-2-600	20.3	12.7	32.7	28.2	6.1
UFFC	35.2	7.0	42.7	12.1	3.0
UFFA-2-600	16.2	4.8	35.5	35.2	8.3

### CO_2_ Adsorption Performance

**Fig. 6:**
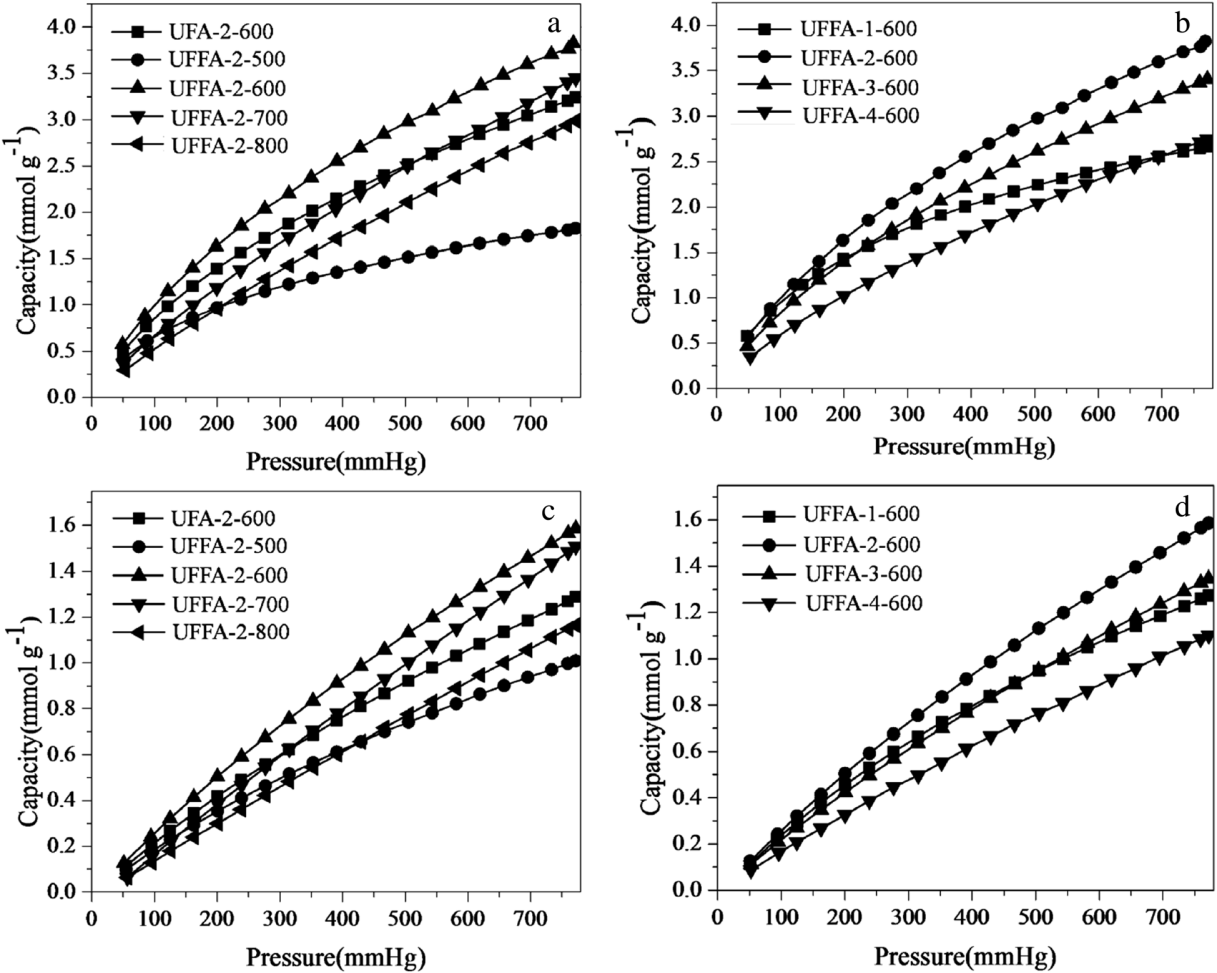
CO_2_ adsorption isotherms of several samples activated at different temperatures and KOH dosages. **a**, **b** Measured at 25 °C and **c**, **d** measured at 75 °C

The adsorption capacities of the different materials were measured at 25 and 75 °C under atmospheric pressure (Fig. 6). The measured adsorption capacities correspond to those expected from their structures and chemical properties. However, the UFFC showed extraordinary uptake of up to 1.66 mmol g^−1^ (Table 3), which is one of the best values reported for carbon-based adsorbents with poor pore structure. In addition, it can be observed that the sample prepared with furfuralcohol (UFFA-2-600) showed an enhanced adsorption capacity compared with UFA-2-600 (3.76 to 3.21 mmol g^−1^). For the samples obtained at different activation conditions, as can be seen in Table 3, the CO_2_ uptakes are in the range of 1.8–3.76 mmol g^−1^ at 25 °C. At a higher temperature of 75 °C, the CO_2_ molecules have higher energy and, hence, can escape from the surface easily. Therefore, the CO_2_ uptakes of the samples are much lower (1.00–1.57 mmol g^−1^) at 75 °C. With the increase of the activation temperature or KOH dosage, the adsorption capacities increased first and then decreased as expected. The sample UFFA-2-600 with high proportion of fine micropore volume and moderate BET surface area showed the highest uptake of about 3.76 mmol g^−1^ at 25 °C, 1 bar. The lower CO_2_ uptakes of the samples activated at mildest conditions (UFFA-2-500 and UFFA-1-600) can be attributed to the lack of activation resulting in poor pore structure. Notably, the sample UFFA-1-600 showed higher CO_2_ uptake than those of UFFA-3-600 and UFFA-4-600 at low pressure (<0.15 bar) although it has poor pore structure, which suggests that the mild activation condition is in favor of the low pressure adsorption. Apparently, there is no obvious relationship between the CO_2_ adsorption capacity and the BET surface area or total pore volume. For instance, the sample UFFA-2-600 showed higher uptake than UFA-2-600, although they have comparable BET surface area and total pore volume.

**Table 3:** CO_2_ capture capacities of various samples at 1 atm and different temperatures

Sample	CO_2_ uptake (mmol g^−1^)	
	25 °C	75 °C
UFC	1.44	
UFFC	1.66	
UFA-2-600	3.21	1.27
UFFA-1-600	2.65	1.26
UFFA-2-600	3.76	1.57
UFFA-3-600	3.37	1.33
UFFA-4-600	2.72	1.09
UFFA-2-500	1.80	1.00
UFFA-2-700	3.40	1.48
UFFA-2-800	2.94	1.15

To determine the effect of the incorporation of nitrogen and the porosity in promoting the adsorption of CO_2_, CO_2_ uptakes have been normalized by micropore volume and N content as shown in Table 4. The uptake per micropore volume decreased when the activation conditions were more severe. For example, the samples UFFA-2-700 and UFFA-2-800 showed high total pore volume but low CO_2_ uptake per micropore volume, indicating that the severe activation conditions have negative effects on the adsorption of CO_2_. However, UFFA-1-600 with low micropore volume showed higher CO_2_ uptake per micropore volume confirming a stronger affinity for CO_2_ molecules, which suggests that the chemical adsorption plays a more important role in enhancing the adsorption of CO_2_. On the contrary, CO_2_ uptakes per millimoles of nitrogen increase with the activation conditions getting more severe. The samples UFFA-2-700 and UFFA-2-800 with well-developed pore structure showed much higher CO_2_ uptakes per millimoles of nitrogen, indicating that the adsorption of CO_2_ on these adsorbents is mainly physical adsorption [32]. However, the UFA-2-600 and UFFA-2-600 samples with comparable CO_2_ uptakes per micropore volume showed different CO_2_ uptakes per millimoles of nitrogen. The sample UFFA-2-600 showed double the CO_2_ uptake per millimoles of nitrogen indicating that its pore structure is more beneficial to CO_2_ adsorption.

**Table 4:** Normalized CO_2_ adsorption capacities by narrow micropore volume and nitrogen content

Sample	Normalized CO_2_ uptake at 25 °C, 1 atm	
	Uptake per micropore volume (mmol CO_2_ cm^−3^)	Uptake per N content (mmol CO_2_ (mmol N)^−1^)
UFA-2-600	7.13	0.32
UFFA-1-600	13.25	0.37
UFFA-2-600	7.67	0.65
UFFA-3-600	6.24	0.61
UFFA-4-600	5.66	0.73
UFFA-2-500	–	0.24
UFFA-2-600	7.67	0.65
UFFA -2-700	4.30	0.76
UFFA-2-800	4.2	1.40

**Fig. 7:**
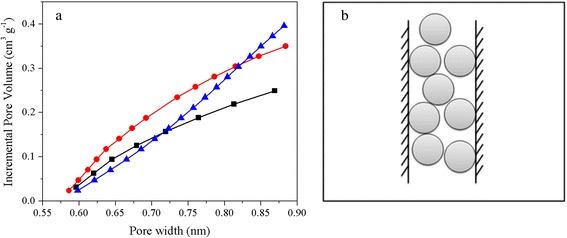
Cumulative pore volume plotted against the pore width for the typical carbons calculated from N_2_ adsorption by the DFT (**a**), *black square* UFA-2-600, *red circle* UFFA-2-600, *blue triangle* UFFA-2-800. Scheme of CO_2_ adsorption in fine micropores (**b**)

The above discussion confirms that CO_2_ adsorption performance of the nitrogen-doped carbon adsorbents is the combination of physical adsorption and chemical adsorption, but the roles they play vary with the changes of pore structures and N contents. As demonstrated in the literatures [33], the physical adsorption mainly occurs in the fine micropores and it could be well described by micropore filling mechanism, especially in ultramicropores (the average pore diameter of ultramicropores is less than 0.7 nm as defined by IUPAC) [34]. The high ultramicropore volume of UFFA-2-600 (0.2 cm^3^ g^−1^) compared with that of UFA-2-600 (0.14 cm^3^ g^−1^) as presented in Fig. 7a is the main reason for the higher CO_2_ uptake in the former. This can also explain the CO_2_ adsorption of the various samples activated at different temperatures or different amounts of KOH. As can be seen in Fig. 2, the micropore volume of UFFA-2-800 is about 0.13 cm^3^ g^−1^ which is much lower than that of UFFA-2-600, resulting in the lower CO_2_ uptake by the former. As shown in the schematic diagram (Fig. 7b), the CO_2_ molecule with a radius of about 0.33 nm can only be filled in the pores by no more than two layers because of their small pore diameter. However, the adsorbed CO_2_ molecules can get condensed and packed in the pores due to the superposition of van der Waals forces given by the two walls because of the small pore diameter of the pores. Based on this view, the CO_2_ uptake is postulated to be closely related to the ultramicropore volume.

**Fig. 8:**
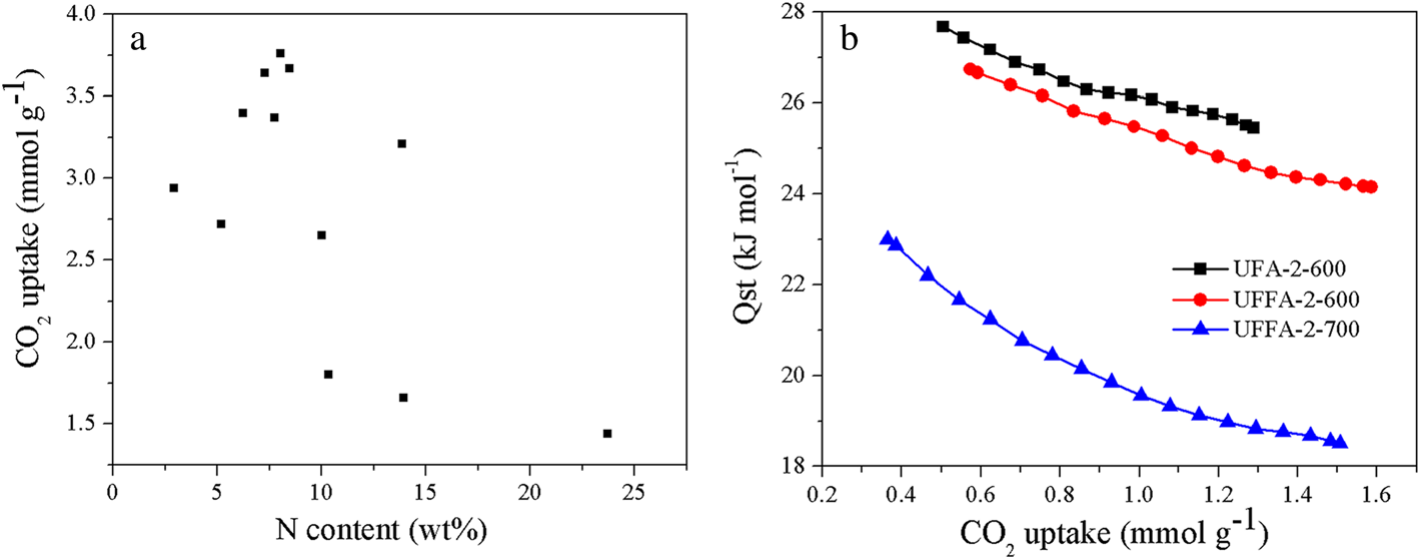
The plot of CO_2_ adsorption capacities vs. N contents of the carbons (**a**). Isosteric heats of CO_2_ adsorption on activated carbons at different CO_2_ uptakes (**b**)

The nitrogen atoms in the pore walls can enhance the effect between the wall and CO_2_ molecule undoubtedly although the mechanism needs further investigation [27, 35, 36]. The high N content and plentiful basic groups is another essential reason for the higher CO_2_ uptake of UFFAs. But as can be seen in Fig. 8a, there is no obvious relationship between the CO_2_ uptake and N content. To further illustrate the effect of nitrogen doping in enhancing the affinity of pore wall, the isosteric heats of adsorption are calculated from the CO_2_ adsorption isotherms at 25 and 75 °C by using the Clausius-Clapeyron equation [37–41]. As can be seen in Fig. 8b, the effect of N doping is reflected by the isosteric heat of adsorption and the isosteric heat of adsorption decreased with the decrease of nitrogen content due to the increase of activation temperature as elaborated above (UFFA-2-600 versus UFFA-2-700). However, the effect of nitrogen in enhancing the capacity is restricted. The sample UFA-2-600 has a lower CO_2_ uptake than that of UFFA-2-600 though it possesses much higher N content (13.92 to 8.03 %). This may be because there is no more space for CO_2_ molecule to be occupied due to the fact that the adsorbed CO_2_ molecules have been packed closely in the ultramicropores as previously described (according to the D-A theory, the CO_2_ molecules fill the micropores in a liquid-like state). The groups containing nitrogen atoms just enhance the interaction between the surface of the adsorbents and CO_2_ molecules, possessing higher adsorption isosteric heat, while the capacity is based on the combination of pore structure and nitrogen content. This may be the main reason why the isosteric heat of adsorption relies on the nitrogen content, but there is no obvious relationship between nitrogen content and adsorption capacity.

## Conclusions

In summary, we have demonstrated a feasible and very simple method for the synthesis of N-doped microporous carbon via a carbonization-activation process by using urea formaldehyde resin co-polymerized with furfuralcohol, which showed tunable surface area and high nitrogen content. N-doped microporous carbon with a high surface area of about 1093 m^2^ g^−1^ and a high density of N functional groups (8.03 %) was obtained. Furfuralcohol plays an important role in improving the pore structure of the adsorbent. More ultramicropores of about 0.6 nm were formed in the above microporous carbon, which is a decisive factor for the CO_2_ adsorption uptake. We also found that much more nitrogen species in the carbonized samples are in the form of nitrogen-containing heterocyclic compounds. The obtained samples showed excellent CO_2_ adsorption uptake in the range of 1.8–3.76 mmol g^−1^ and 1.00–1.57 mmol g^−1^ at 25 and 75 °C, respectively, confirming the combination of physical and chemical adsorption mechanism on CO_2_ adsorption capacity improvement. In addition, this reaction system can be further improved using other carbon sources.
